# Concomitant TP53 mutation in early-stage resected EGFR-mutated non-small cell lung cancer: a narrative approach in a genetically admixed Brazilian cohort

**DOI:** 10.1590/1414-431X2023e12488

**Published:** 2023-04-07

**Authors:** J. Machado-Rugolo, C.M. Baldavira, T.G. Prieto, E.H.R. Olivieri, A.T. Fabro, C.A. Rainho, E.C. Castelli, P.E.M. Ribolla, A.M. Ab'Saber, T. Takagaki, M.A. Nagai, V.L. Capelozzi

**Affiliations:** 1 Laboratório de Histomorfometria e Genômica Pulmonar, Departamento de Patologia, Faculdade de Medicina, Universidade de São Paulo, São Paulo, SP, Brasil; 2 Centro de Avaliação de Tecnologias em Saúde, Hospital das Clínicas da Faculdade de Medicina de Botucatu, Universidade Estadual Paulista, Botucatu, SP, Brasil; 3 Centro Internacional de Pesquisa/CIPE, AC Camargo Cancer Center, São Paulo, São Paulo, SP, Brasil; 4 Departamento de Patologia e Medicina Legal, Laboratório de Medicina Respiratória, Faculdade de Medicina de Ribeirão Preto, Universidade de São Paulo, Ribeirão Preto, SP, Brasil; 5 Instituto de Biociências, Departamento de Ciências Químicas e Biológicas, Universidade Estadual Paulista, Botucatu, SP, Brasil; 6 Laboratório de Genética Molecular e Bioinformática, Unidade de Pesquisa Experimental, Faculdade de Medicina, Universidade Estadual Paulista, Botucatu, SP, Brasil; 7 Departamento de Patologia, Faculdade de Medicina, Universidade Estadual Paulista, Botucatu, SP, Brasil; 8 Instituto de Biotecnologia, Universidade Estadual Paulista, Botucatu, SP, Brasil; 9 Instituto de Biociências, Departamento de Bioestatística, Biologia Vegetal, Parasitologia e Zoologia, Universidade Estadual Paulista, Botucatu, SP, Brasil; 10 Divisão de Pneumologia, Instituto do Coração, Faculdade de Medicina, Universidade de São Paulo, São Paulo, SP, Brasil; 11 Departamento de Radiologia e Oncologia, Faculdade de Medicina, Universidade de São Paulo, São Paulo, SP, Brasil; 12 Laboratório de Genética Molecular, Centro de Pesquisa Translacional em Oncologia, Instituto do Câncer de São Paulo, São Paulo, SP, Brasil

**Keywords:** Non-small-cell lung cancer (NSCLC), Epidermal growth factor receptor (EGFR), Tumor protein 53 (TP53), Mutation, Survival

## Abstract

*TP53* mutations are frequent in non-small cell lung cancer (NSCLC) and have been associated with poor outcome. The prognostic and predictive relevance of *EGFR*/*TP53* co-mutations in NSCLC is controversial. We analyzed lung tissue specimens from 70 patients with NSCLC using next-generation sequencing to determine *EGFR* and *TP53* status and the association between these status with baseline patient and tumor characteristics, adjuvant treatments, relapse, and progression-free (PFS) and overall survival (OS) after surgical resection. We found the *EGFR* mutation in 32.9% of patients (20% classical mutations and 12.9% uncommon mutations). *TP53* missense mutations occurred in 25.7% and *TP53/EGFR* co-mutations occurred in 43.5% of patients. Stage after surgical resection was significantly associated with OS (P=0.028). We identified an association between progression-free survival and poor outcome in patients with distant metastases (P=0.007). We found a marginally significant difference in OS between genders (P=0.057) and between mutant and wild type *TP53* (P=0.079). In univariate analysis, distant metastases (P=0.027), pathological stage (IIIA-IIIB *vs* I-II; P=0.028), and *TP53* status (borderline significance between wild type and mutant; P=0.079) influenced OS. In multivariable analysis, a significant model for high risk of death and poor OS (P=0.029) selected patients in stage IIIA-IIIB, with relapse and distant metastases, non-responsive to platin-based chemotherapy and erlotinib, with tumors harboring *EGFR* uncommon mutations, with *TP53* mutant, and with *EGFR/TP53* co-mutations. Our study suggested that *TP53* mutation tends to confer poor survival and a potentially negative predictive effect associated with a non-response to platinum-based chemotherapy and erlotinib in early-stage resected *EGFR*-mutated NSCLC.

## Introduction

In 2020, lung cancer was reported to be the main cause of cancer-related deaths and the second more prevalent diagnosed malignancy (1). The most frequent type of lung cancer is non-small cell lung cancer (NSCLC), which accounts for 85-90% of lung cancer patients (2). At the first clinical consultation, most patients already present advanced stages of the disease, so the available treatments are chemotherapy, immunotherapy, and target therapy.

Particularly, the outcomes of lung adenocarcinomas have improved due to the increasing characterization of oncogenic drivers and the possibility of efficiently targeting these drivers (3). This advance mainly relates to the development of minor-molecule tyrosine kinase inhibitors (TKI) targeting the activating mutations in the epidermal growth factor receptor (*EGFR*) gene (4). In patients with advanced disease, EGFR TKIs have shown satisfactory objective response rates and prolonged progression-free survival (PFS) compared with chemotherapy (5).

However, approximately 30% of patients develop primary resistance to TKIs and/or chemotherapy, and the disease eventually relapses months to years after starting TKIs and chemotherapy. Relatively few studies have been conducted on the mechanism of treatment resistance. Uncommon and multiple somatic mutations have been associated with worse outcomes compared with tumors with a single classical mutation (6,7). Access to next generation sequencing (NGS) allows detection of co-mutations in advanced *EGFR* mutated-NSCLC patients. This detection suggests that these co-mutations might be one of the mechanisms of drug resistance, among which *TP53* mutations were the most frequent co-mutations in all types of lung cancer (8). Some clinical studies suggest a negative prognostic effect of *TP53* mutations on NSCLC with adjuvant chemotherapy in patients with completely resected *TP53*-mutant NSCLC (9). Unfortunately, to date, there are no approved drugs that specifically target *TP53* in NSCLC.

Since lung cancer investigation has focused basically on Caucasian and Asian cohorts (2), we know little about the *EGFR*/*TP53* co-mutations of NSCLC in the Brazilian population. Current research indicates that race plays a role in the genomics of NSCLC in this population. For example, in patients with adenocarcinoma, the estimated prevalence of EGFR mutations in Asians is around 60%, while it is only 20% percent in Caucasians (4). In the limited series of Brazilian patients reported, we observed a considerable variation in mutation rate in the south of Brazil (19%) and 22-30% in the southeast (particularly in the city of São Paulo). This variation in mutation rate suggests an increased prevalence of Amerindian and Asian ancestries (10). However, there is no information about EGFR/P53 co-mutations in Brazilian patients with NSCLC.

Defining of the combined molecular pathogenesis of NSCLC is crucial in Brazilians, as this population has both a higher incidence of NSCLC and an increased mortality from the disease compared with Caucasians and Asians and eventually greater resistance to adjuvant treatment. Thus, we designed the present study to evaluate the prognostic and predictive value of *EGFR/TP53* co-mutation detected by NGS in surgically resected NSCLC patients. We aimed to provide a narrative portrait of the effect of the co-mutation on PFS and overall survival (OS) in patients receiving adjuvant treatment.

## Material and Methods

### Patient population and data collection

Because the new *EGFR/TP53* co-mutation can also arise from sequencing artifacts, especially artifacts associated with formalin-related DNA damage, as described in Wong et al. (11), we conducted our investigation in fresh-frozen specimens from Brazilian patients with lung cancer. Specimen collection took place during surgical resections conducted from August 2003 to August 2010 at the A.C. Camargo Cancer Center, a tertiary referral center for the treatment of lung cancer in São Paulo, Brazil. Our group obtained a total of 70 fresh-frozen specimens from Brazilian patients with lung cancer from different regions of the country. Two experienced lung pathologists reviewed the histologic diagnoses, assessed the accuracy of the histologic diagnoses based on the World Health Organization (WHO) 2021 classification system (12), and stratified them into non-squamous non-small lung cancer (n=46) and squamous non-small cell lung cancer (n=24). *EGFR* and *TP53* statuses were correlated with baseline characteristics, including age, sex, ethnicity, smoking history, pathologic TNM stage (13), histology, type of *EGFR* mutation, radiotherapy, platinum-based chemotherapy, tyrosine kinase inhibitors (TKI), and relapse free survival (RFS) and overall survival (OS) after primary surgical resection, relapse, and development of distant metastases.

The study was approved in accordance with the ethical standards of the local committee on human experimentation (Research Ethics Committee of University of São Paulo Medical School - CAAE: 79769017.1.0000.5440; opinion number: 2.673.320). The informed consent was waived due to the retrospective study design and the identity of the subjects was omitted and anonymized.

### Targeted gene profiling

The DNA of fresh tumor tissue was extracted using the QIAamp DNA Mini Kit (Qiagen, Germany), according to the manufacturer's recommendations, and quantified using the Qubit^®^ 3.0 Fluorometer (Invitrogen, Life Technologies, USA). The *EGFR* and *TP53* genes were targeted using TruSeq Custom Amplicon Panel v1.5 kit (TSCAP, Illumina, USA), followed by massive parallel sequencing on an Illumina MiSeq platform consisting of 150 bp paired-end reads (300 cycles). All tumor specimens had an average sequencing depth of the target region ≥100× and coverage of the target region >90% at 30×. The Molecular Genetics and Bioinformatics Laboratory of the Experimental Research Unit (UNIPEX) at the Medical School of São Paulo State University (UNESP) performed the sequencing data analyses to reduce the effects of PCR amplification and sequencing artifacts. The raw sequencing data were base-called and demultiplexed using MiSeq Reporter v.1.8.1 (Illumina) with default parameters, and FastQC files were generated for downstream data analysis. Filtered reads were aligned to the human genome (hg19, GRCh37) using the Burrows-Wheeler Alignment tool (BWA) v.0.7.10 (http://bio-bwa.sourceforge.net). After alignment, the SAMtools software (https://www.htslib.org/) was applied to convert the alignment files to an indexed binary alignment map format. The single nucleotide variants (SNVs) and short insertions and deletions (INDELs) were named using the GATK UnifiedGenotyper, including HaplotypeCaller with default parameters based on hg19 and annotated with dbSNP version 144 (gatk.broadinstitute.org). Our group used the following cut-off criteria to reduce false-positive somatic mutations that might originate from germline variants: number of reads with the altered base in the tumor ≥10, mutations detected at a position of total read depth of ≥100, frequency of the reads with the altered base in the tumor ≥5% except for variants also reported in the COSMIC database, minor allele frequency <0.1% in two publicly available databases, namely 1000 Genomes and Exome Aggregation Consortium. We annotated the variants using the VEP software (grch37.ensembl.org) based on the consequences, predicted impacts, and reported allele frequencies in the population. The variants of unknown significance (VUS) were checked on the ClinVar database (http://www.ncbi.nlm.nih.gov/clinvar/).

### Statistical analysis

We conducted the Pearson chi-squared test or Fisher's exact test to compare categorical variables and the one-way analysis of variance to compare continuous variables. OS definition - the primary end point - was defined as the time from surgery to death from any cause or to the last follow-up of surviving patients. We set the PFS as the time from surgery to recurrence or death from any cause or to the last follow-up of surviving patients. As our cases were from a genetically mixed population, we evaluated the prognostic and predictive value of *TP53*/*EGFR* co-mutation status in non-squamous NSCLC and squamous NSCLC. Hazard ratios (HRs) and their confidence intervals (CIs) were estimated via a multivariable Cox proportional hazards model including the core variables with P<0.1 in the univariate analysis: gender, pathological TNM stage, relapse, and adjuvant treatment (chemotherapy and radiotherapy), *TP53* status, and *EGFR*/*TP53* co-mutation. We set the statistical significance at P<0.05. Survival curves were based on Kaplan-Meier methods and presented with unadjusted HRs from the Cox model and P*-*values using log rank statistic. The SPSS software version 22.0 (IBM Corporation, USA) was used for the statistical analyses.

## Results

### Clinicopathological characteristics

Overall, 70 patients with clinical stage for NSCLC surgical resection were included in the study; however, some patients lacked follow-up information. There were 44 males (62.9%) and 21 females (30%) with a median age of 65 years (range=41-96 years). According to ancestry, there were 48 European (68.6%), 2 Asian (2.9%), and 2 African (2.9%) patients. Twenty-one patients (30%) were current smokers. Samples were stratified into non-squamous NSCLC in 46 cases (65.7%) and squamous NSCLC in 24 cases (34.3%). After surgical resection, tumor staging was as follows: IA (14/20%), IB (10/14.3%), IIA (10/14.3%), IIB (13/18.6%), IIIA (11/15.7%), and IIIB (3/4.3%). During follow-up, locoregional relapse and distant metastases each occurred in 12 patients (17.4%). Metastases in the central nervous system and bone were the most common (4/5.7% and 4/5.7%), followed by liver and kidney metastases (2/2.8 and 2/2.8%). Adjuvant treatment included platinum-based chemotherapy in 31 (44.3%), radiotherapy in 4 (4.3%), chemoradiotherapy in 6 (8.6%), and TKI in 2 (2.9%) patients. At the last follow-up, 38 (54.3%) patients had died (Table 1).

**Table 1:** Frequency of demographic and clinical characteristics of 70 non-small cell lung cancer (NSCLC) patients.

Characteristics	Number of patients (n=70)
Age, years	
Median (range)	65 (41-96)
≤77	32 (45.7%)
>77	33 (47.1%)
Gender	
Male	44 (62.9%)
Female	21 (30.0%)
Ancestry	
European	48 (68.6%)
Asian	2 (2.9%)
African	2 (2.9%)
Smoking status	
Smoker	21 (30.0%)
Non-smoker	7 (10.0%)
Histological subtype	
Non-squamous NSCLC	46 (65.71%)
Squamous NSCLC	24 (34.3%)
Pathological TNM stage^†^	
IA	14 (20.0%)
IB	10 (14.3%)
IIA	10 (14.3%)
IIB	13 (18.6%)
IIIA	11 (15.7%)
IIIB	3 (4.3%)
Relapse	
No	13 (18.6%)
Locoregional	12 (17.4%)
Distant metastasis	12 (17.4%)
Central nervous system	4 (5.7%)
Bones	4 (5.7%)
Liver	2 (2.8%)
Kidney	2 (2.8%)
Adjuvant therapy	
Chemotherapy platinum-based	31 (44.3%)
Radiotherapy	4 (4.3%)
Chemoradiotherapy	6 (8.6%)
Tyrosine kinase inhibitor (erlotinib)	2 (2.9%)
Status for overall survival	
Alive	23 (32.9%)
Dead	38 (54.3%)
Follow-up (months)	49 (0-175)
*EGFR* status	
Classic mutation (18-21 exons)	14 (20.0%)
Uncommon mutation	9 (12.9%)
Wild type	47 (67.1%)
*TP53* status	
Missense mutation	18 (25.7%)
Others	10 (14.2%)
Wild type	41 (58.6%)
*EGFR*/*TP53* dual mutation	
Yes	10 (43.5%)
No	13 (56.5%)

Data are reported as number and percentage. Some cases lacked follow-up information: age (5); gender (5); race (18); smoking status (42); TNM stage (9); Status (9). ^†^8th Edition International Association for the Study of Lung Cancer (Ref. 13; doi: 10.1016/j.jtho.2015.09.009). NSCLC, non-small cell lung cancer; *TP53*: tumor protein p53; *EGFR*: epidermal growth factor receptor.

### Mutation status

In our cohort, the mutation rate of *TP53* was 41.5% (n=29/70) and of *EGFR* was 32.9% (n=23/70), being that the well-established *EGFR* mutation in exons 18-21 was 20% (n=14/70) and in other exons was 12.9% (n=9/70). From the mutated *EGFR* cohort, *EGFR/TP53* co-mutation was found in 10 patients (n=10/23; 43.5%), as shown in Table 1. The genomic profile of the *TP53* gene identified is summarized in Table 2. It is worth noting that, the majority of *TP53* mutations was missense and of pathogenic significance. Tables 3 and 4, respectively, show the association between patient characteristics and *EGFR* and *TP53* status. *EGFR* uncommon mutations were most common in male patients (P=0.005). There were also significantly more non-squamous NSCLC (P=0.035) with locoregional relapse (P=0.028) in the *TP53* wild-type group. *EGFR/TP53* co-mutation did not differ among the clinicopathological characteristics of the patients. Finally, we examined the importance of the *TP53* mutation identified in *EGFR*-mutant NSCLC patients stratified in *EGFR* co-existing pathogenic mutation (N=5) and *EGFR* co-existing VUS (N=5) (Supplementary Table S1). Clinicopathological characteristics of NSCLC patients with *EGFR/TP53* co-mutation are shown in Supplementary Table S2. Overall, dual *TP53/EGFR* mutations were found in 10 patients (43.5%), 8 males and 2 females, with a median age of 76 years, mostly from European ancestry, and in early disease stage (n=9). Common clinical characteristics of patients whose tumors harbored *TP53* mutation in *EGFR* co-existing pathogenic mutation were older patients with non-squamous NSCLC. These patients developed distant metastases after surgical resection with partial response to systemic chemotherapy and EGFR-TKI and short survival. In contrast, most patients with squamous NSCLC harboring *TP53* and coexistent *EGFR* VUS mutation were younger, without tumor relapse, received no adjuvant chemotherapy, and progressed with long survival.

**Table 2:** Spectrum of TP53 mutations identified in a Brazilian NSCLC cohort by Next Generation Sequencing.

ID variant	Genomic position*	HGVS nucleotide	HGVS protein	Variant type	Molecular consequence	Clinical significance**	Frequency
rs397516435	7578263	c.586C>T	p.Arg196Ter	Stop gained	Nonsense	Pathogenic	1
COSM48979	7577586	c.692_694del	p.Thr231del	Deletion	Deletion-In frame	Pathogenic	1
rs137852789	7578470	c.460G>A	p.Gly154Ser	Missense	Missense	Uncertain significance	1
COSM5315967	7578469	c.461delG	p.Gly154Afs*16	Deletion	Deletion-Frameshift	Not provided	1
rs1057520007	7578235	c.614A>C	p.Tyr205Ser	Missense	Missense	Likely pathogenic	1
rs746791390	7579594	c.97-4A>G	-	Splice region	-	Likely benign	1
rs587780074	7577544	c.737T>A	p.Met246Lys	Missense	Missense	Likely pathogenic	1
rs148924904	7578442	c.488A>G	p.Tyr163Cys	Missense	Missense	Pathogenic	1
rs28934576	7577120	c.818G>T	p.Arg273Leu	Missense	Missense	Pathogenic	2
rs587782082	7577536	c.745A>G	p.Arg249Gly	Missense	Missense	Uncertain significance	1
rs866380588	7578275	c.574C>T	p.Gln192Ter	Stop gained	Nonsense	Pathogenic	1
rs1057519991	7578394	c.536A>C	p.His179Pro	Missense	Missense	Conflicting interpretations of pathogenicity	2
rs730882001	7578437	c.493C>T	p.Gln165Ter	Stop gained	Nonsense	Pathogenic	1
rs28934575	7577548	c.733G>T	p.Gly245Cys	Missense	Missense	Pathogenic	3
rs28934571	7577534	c.747G>C	p.Arg249Ser	Missense	Missense	Uncertain significance	1
COSM44478	7577557	c.716_724del	p.N239_C242delinsS	Deletion	Deletion-In frame	Uncertain significance	1
rs1057520000	7578478	c.452C>G	p.Pro151Arg	Missense	Missense	Pathogenic	1
rs193920774	7577141	c.797G>A	p.Gly266Glu	Missense	Missense	Pathogenic/Likely pathogenic	1
rs867114783	7578427	c.503A>G	p.His168Arg	Missense	Missense	Conflicting interpretations of pathogenicity	1
rs1131691035	7578257	c.592delG	p.Glu198fs	Deletion	Deletion-Frameshift	Pathogenic	1
rs587780070	7578395	c.535C>T	p.His179Tyr	Missense	Missense	Pathogenic/Likely pathogenic	1
rs11540652	7577538	c.743G>T	p.Arg248Leu	Missense	Missense	Pathogenic	1
COSM6965992	7578466	c.432_463del	p.Q144Hfs*26	Deletion	Deletion-Frameshift	Pathogenic	1
rs121912664	7574017	c.1010G>A	p.Arg337His	Missense	Missense	Pathogenic/Likely pathogenic	1
COSM11354	7576855	c.991C>T	p.Gln331Ter	Stop gained	Nonsense	Pathogenic	1

TP53: Tumor protein p53; NSCLC: non-small cell lung cancer*;* rs: reference single nucleotide polymorphism; COSM: Catalogue of somatic mutations in cancer; HGVS: Human Genome Variant Society. *Genome Reference Consortium Human Build 37 (GRCh37; hg19). **ClinVar (http://www.ncbi.nlm.nih.gov/clinvar/).

**Table 3:** Clinicopathological characteristics of 70 patients with non-small cell lung cancer (NSCLC) stratified according to *EGFR* mutational status using Pearson's chi-squared test.

Characteristics	*EGFR* status			
	Classic mutations (18-21 exons)	Uncommon mutations	Wild type	P-value
Age (median in years)				0.938
≤65	3 (4.6%)	8 (12.3%)	21 (32.3%)	
>65	4 (6.2%)	8 (12.3%)	21 (32.3%)	
Gender, n (%)				0.005
Male	1 (1.5%)	13 (20.0%)	30 (46.2%)	
Female	6 (9.2%)	3 (4.6%)	12 (18.5%)	
Ancestry				0.573
European	7 (13.5%)	8 (15.4%)	33 (63.5%)	
Asian	0 (0.0%)	1 (1.9%)	1 (1.9%)	
African	0 (0.0%)	1 (1.9%)	1 (1.9%)	
Smoking status				0.206
Yes	1 (3.6%)	5 (17.9%)	15 (53.6%)	
No	2 (7.1%)	1 (3.6%)	4 (14.2%)	
Histology				0.341
Non-squamous NSCLC	7 (7.1%)	11 (14.4%)	28 (40.1%)	
Squamous NSCLC	0 (0.0%)	5 (7.1%)	19 (27.1%)	
Pathological Stage^†^				0.178
I-II	3 (4.7%)	13 (20.3%)	29 (45.2%)	
IIIA-IIIB	4 (6.3%)	3 (4.7%)	12 (18.8%)	
Treatment				0.371
Chemotherapy	3 (11.1%)	7 (25.9%)	9 (33.3%)	
Radiotherapy	1 (3.7%)	6 (21.4%)	2 (7.4%)	
Chemoradiotherapy	0 (0.0%)	0 (0.0%)	3 (11.1%)	
Erlotinib	0 (0.0%)	0 (0.0%)	2 (7.4)	
Relapse				0.339
No	2 (5.9%)	3 (8.8%)	8 (23.5%)	
Locoregional	2 (5.9%)	4 (11.8%)	4 (11.8%)	
Distant with CNS metastases	0 (0.0%)	2 (5.9%)	9 (26.4%)	
Status				0.955
Alive	3 (4.9%)	6 (9.8%)	14 (23%)	
Dead	4 (6.6%)	10 (16.4%)	24 (39.3%)	

Data are reported as number and percentage. ^†^8th International Association for the Study of Lung Cancer (Ref. 13; doi: 10.1016/j.jtho.2015.09.009).

**Table 4:** Clinicopathological characteristics of 70 patients with NSCLC stratified according to *TP53* status using Pearson's chi-squared test.

Characteristics	*TP53* wild-type	*TP53* mutant	P-value
Age (median in years)			0.550
≤65	12 (30.8%)	7 (17.9%)	
>65	12 (30.8%)	8 (20.5%)	
Gender, n (%)			0.159
Male	17 (42.5%)	7 (17.5%)	
Female	8 (20%)	8 (20%)	
Ancestry			
European	28 (51.9%)	22 (40.7%)	0.804
Asian	2 (3.7%)	0 (0%)	
African	1 (1.8%)	1 (1.8%)	
Smoke status			0.109
Yes	16 (22.9%)	10 (14.2%)	
No	25 (30.7%)	19 (27.1%)	
Histology			0.035
Non-squamous NSCLC	31 (44.3%)	15 (21.4%)	
Squamous NSCLC	10 (14.3%)	14 (20.0%)	
Pathological stage^†^			0.719
I-II	36 (51.4%)	26 (37.1%)	
IIIA-IIIB	5 (7.1%)	3 (4.3%)	
EGFR status			0.588
Wild type	27 (38.6%)	19 (27.1%)	
Mutant	14 (20.0%)	10 (14.3%)	
Treatment			0.770
Chemotherapy	11 (40.7%)	6 (22.2%)	
Radiotherapy	3 (11.1%)	1 (3,7%)	
Chemoradiotherapy	2 (7.4%)	1 (3.7%)	
Erlotinib	2 (7.4%)	0 (0.0%)	0.05
Relapse			0.028
Free	6 (17.6%)	6 (17.7%)	
Locoregional	17 (47.2%)	4 (11.1%)	
Distant CNS metastases	0 (0.0%)	8.3 (0.0%)	
Status			0.955
Alive	7 (25.0%)	6 (21.4%)	
Dead	12 (42.9%)	3 (10.7%)	

Data are reported as number and percentage. ^†^8th International Association for the Study of Lung Cancer (Ref. 13; doi: 10.1016/j.jtho.2015.09.009). NSCLC: Non-small cell lung cancer; CNS: central nervous system.

### Survival analysis

**Figure 1:**
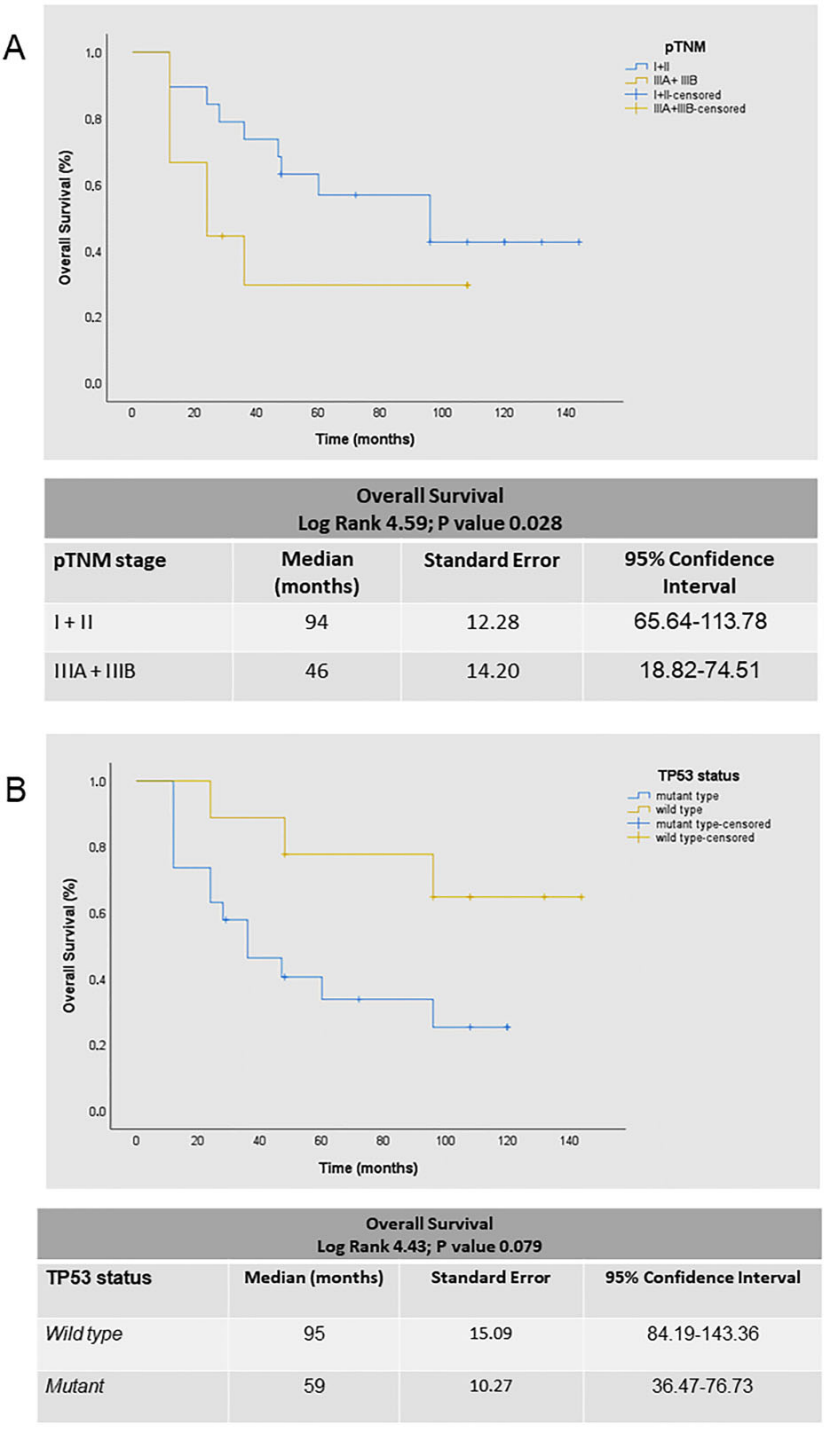
Overall survival (OS) in non-small cell lung cancer (NSCLC) patients. The univariate and multivariate analyses employed a Cox proportional hazards model with chi-squared 15.60, P=0.029. Kaplan-Meier curves according to (**A**) OS in patients with different pathologic stages (pTNM). Stage after surgical resection was significantly associated with OS. **B**, OS in patients with different *TP53* mutation status. Among surgically resected patients, there was a difference of marginal significance in OS for *TP53* mutant *vs* wild-type.

**Figure 2:**
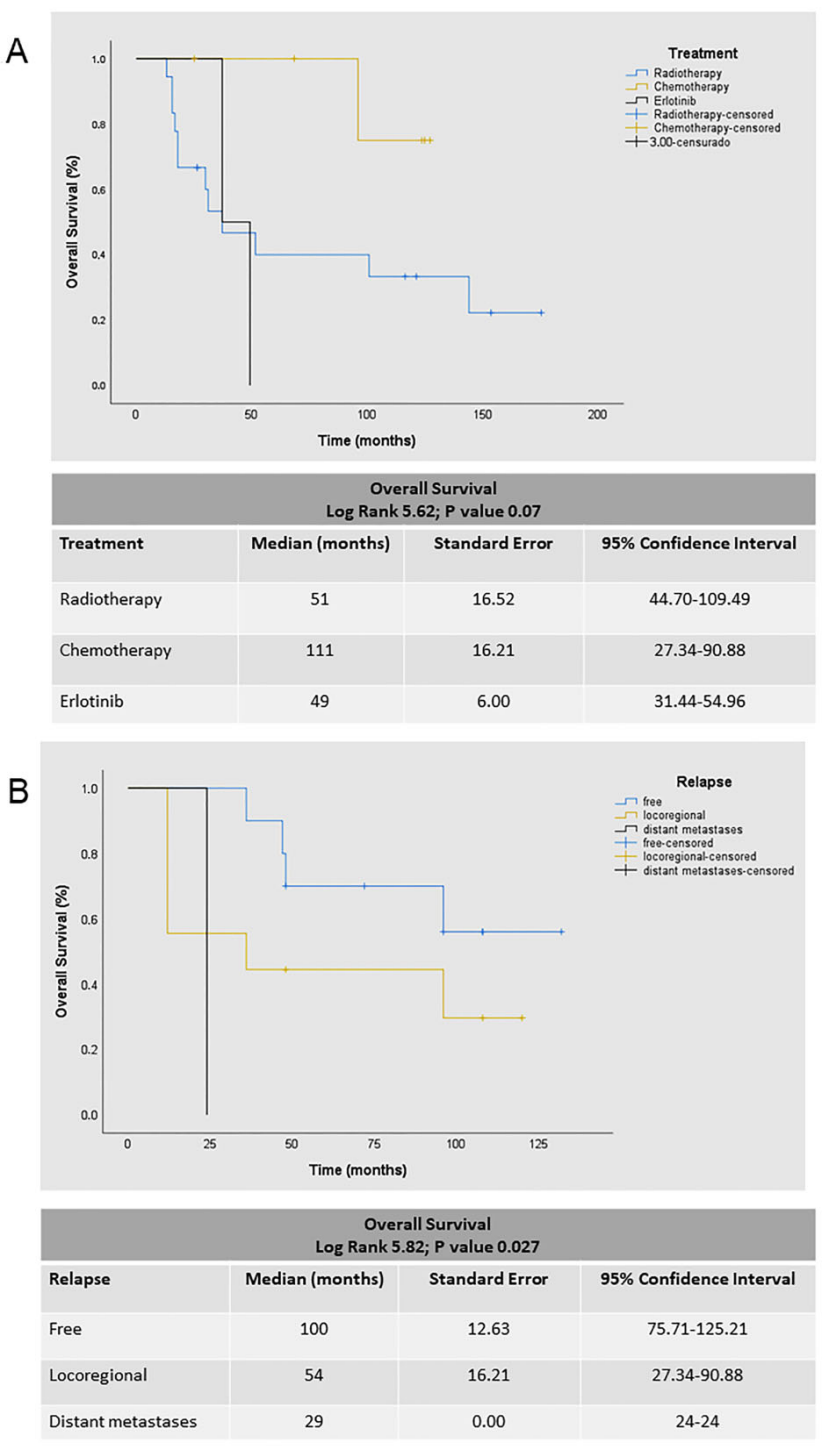
Overall survival (OS) in non-small cell lung cancer (NSCLC) patients. The univariate and multivariate analyses employed a Cox proportional hazards model with chi-squared 15.60, P=0.029. **A**, OS in patients with different adjuvant treatment. There was difference of marginal significance for *TP53* status in OS for the subset of patients who received adjuvant chemotherapy. **B**, OS in patients with or without relapse. OS was associated with poor outcomes in patients with distant metastases.

Preliminary examination of Kaplan-Meier survival curves demonstrated that patients with pathological stages IA, IB, IIA, and IIB had approximately the same hazard for survival, with a median survival time of 85 months. Thus, we coded overall pathological stage as a single dummy variable with a value of 1 for stages I and II and a value of 2 for stages IIIA and IIIB. The results of the Cox model analysis are reported in Table 5. Among the entire cohort of 70 patients, there were 38 deaths (54.3%). For the overall sample, in the univariate analysis, stage after surgical resection (IIIA *vs* I-II; P=0.028; Figure 1A), *TP53* status (borderline for wild type *vs* mutant; P=0.079: Figure 1B), and EGFR/TP53 co-mutation status (borderline for wild type *vs* mutant; P=0.061) influenced OS. We also assessed the effect of relapse and *TP53* status on OS in the subset of patients who received adjuvant chemotherapy (n=24; 12 mutants *vs* 12 wild types) and had metastases (n=19; 16 locoregional *vs* 3 distant), and we identified a borderline significance (P=0.07 and P=0.06; Figure 2A and B, respectively). It is worth noting that the abrupt drop of survival curves (black line) in both Figure 2A and B refers to three patients in advanced stage with brain metastases, two of which were treated with erlotinib, therefore reflecting the small number of patients. In multivariate analysis, the significant factors for the high risk of death model (P=0.029) were stage IIIA-IIIB, relapse with distant metastases, non-response to chemotherapy, tumors harboring *EGFR* uncommon mutations, *TP53* mutation, and *EGFR/TP53* co-mutations.

**Table 5:** Variables associated with overall survival (OS) in non-small cell lung cancer (NSCLC) patients.

Clinicopathological characteristics	OS (months)	Univariate analysis				Multivariate analysis	
		HR (95%CI)	HR	P-value		HR (95%CI)	P-value
Age (median in years): ≤65 *vs* >65	98 *vs* 71	0.67 (0.30-1.48)	-0.39	0.32			
Gender							
Male *vs* Female	61 *vs* 102	2.34 (0.97-5.63)	0.85	**0.057**			
Ancestry							
European	82	0.26 (0.05-1.20)	-1.34	0.73			
Asian	101	0.23 (0.02-2.77)	-1.43	0.25			
African (reference)	25	1		0.22			
Smoking status							
No *vs* Yes	72 *vs* 80	0.56 (0.12-2.59)	-0.58	0.45			
Pathological stage^†^							
I-II	94	0.70 (0.31-1.58)	-0.34	**0.028**		1.53 (0.36-6.47)	0.56
IIIA-IIIB (reference)	46	1				1	
Relapse							
No	100	0.15 (0.04-0.61)	-1.84	**0.007**		0.04 (0.004-0.43)	**0.008**
Locoregional	59	0.68 (0.25-1.86)	-0.37	0.46		0.78 (0.15-3.92)	0.77
Distant metastasis (reference)	24	1		**0.027**		1	**0.022**
Histological subtypes							
Non-squamous NSCLC	87	1.21 (0.38-3.84)	0.19	0.73			
Squamous NSCLC (reference)	52	1		0.13			
Adjuvant therapy							
Chemotherapy	111	1.06 (0.23-4.76)	0.06	**0.07**		1.20 (0.21-6.72)	0.82
Radiotherapy	51	0.79 (0.25-2.45)	-0.23	0.68		0.48 (0.12-1.92)	0.30
Erlotinib (reference)	49	1		0.59		1	0.48
EGFR mutation status							
Classic mutations (18-21 exons)	66	1.84 (0.54-6.27)	0.61	0.32		1.421 (0.271-7.466)	0.67
Uncommon mutation (others exons)	61	0.90 (0.37-2.16)	-0.10	0.81		0.58 (0.13-2.60)	0.48
Wild-type (reference)	81	1				1	0.93
TP53 mutation status							
Wild-type	95	0.595 (0.077- 4.614)	0.520	**0.079**		1.256 (031-5.799)	0.64
Mutant (reference)	59	1				1	
EGFR/TP53 co-mutation status							
Wild-type	90	0.586 (0.196-1.746)	0.535	**0.061**		0.60 (0.07-4.99)	0.63
Mutant (reference)	48	1				1	

A Cox proportional hazards model was used for the univariate and multivariate analyses (chi-squared 15.60, P=0.029). Bold type indicates statistical significance. ^†^8th International Association for the Study of Lung Cancer (Ref. 13; doi: 10.1016/j.jtho.2015.09.009).

## Discussion

The narrative portrait of our study population showed that the main reason for the failure of surgical resection and adjuvant treatment in prolonging survival of patients with early-stage NSCLC was that routine pathological analysis failed to predict relapse and metastases. In fact, during follow-up, even the patients in early stage developed locoregional and distant relapse with central nervous system metastases. These patients received adjuvant radiotherapy and chemotherapy. Nevertheless, the mortality rate was 54.3%. Lung cancer is a genetic disease that results from a multistep process involving genetic and epigenetic changes, especially activation of growth pathways and inhibition of tumor suppressor pathways. Therefore, the relevant question is whether supplementary genetic information from tumor tissue combined with classic TNM stage classification can help us to improve risk stratification and patient selection for personalized treatment.

It is evident that lung cancer investigation and treatment have entered an era of personalized medicine, which uses biomarkers to stratify patients who are more likely to benefit from a specific drug. However, not all NSCLC are phenotypically equal, and some drugs that are effective against non-squamous NSCLC, including pemetrexed, are ineffective in squamous NSCLC, while others, like bevacizumab, are potentially dangerous (14,15). Personalized treatment is more advanced in adenocarcinoma, for which patients are routinely investigated for oncogenic drivers to select therapy. EGFR target therapy was approved as the standard of care for patients with classical *EGFR* mutations (exon 19 deletions), but 20-30% of these patients developed primary resistance to EGFR target therapy (16). To improve the therapeutic outcome of these patients, it is crucial that we understand the mechanisms underlying resistance. Uncommon *EGFR* mutations, such as the exon 20 insertion mutation, could result in primary resistance to EGFR target therapy (17). The advent of NGS created the possibility to detect co-mutations in *EGFR-*mutated NSCLC patients. These co-mutations might be one of the mechanisms of primary drug resistance, among which *TP53* mutations were the most frequent co-mutations (3).

In the present study, we evaluated the clinical outcomes of patients with *EGFR*-driven early NSCLC based on their *TP53* mutational status. In our population, *EGFR* mutation was found in 32.9% of patients. However, mutations in exons 18-21 were 20%, with the most common mutations being short deletions in exon 19 (E746-A750) (18). These findings are consistent with previous works, showing *EGFR* mutations as the predominant driver mutations in patients with NSCLC (19). In addition, classical mutations such as an exon 19 short deletion and an exon 21 point mutation, L858R, are the most common mutations, accounting for about 85-90% (20,21).

*TP53* gene, located on the short arm of chromosome 17 (17p13), is involved in many biological processes, including DNA repair, metabolism, cell cycle arrest, apoptosis, and aging (22). In our population, about 40% of patients harbored *TP53* mutations. We found *TP53* mutations in 21.4% of non-squamous cell carcinomas and 20% of squamous cell carcinomas, contrasting with studies reporting 40 and 51%, respectively (23). In the current study, *TP53/EGFR* co-mutation was found in 43.5% of patients with early-stage NSCLC. Previous reports indicated that 17-72% of advanced *EGFR-*mutant lung cancers harbor *TP53* mutations (3,22). Co-mutation status did not differ by age, gender, smoking history, histotypes, and pathologic stage, as previously reported by the LACE-Bio group (22).

Currently, the Brazilian population is one of the most genetically diverse populations in the world. Such diversity results from five centuries of admixture between four ethnic groups: Asian, European, African, and Amerindian. Despite the shortage of information about ancestry in our population, the European ancestry had a higher proportion of classical and uncommon *EGFR* and *TP53* mutations than the Asian and African ancestries, whereas *EFGR/TP53* co-mutations occurred in 16.7% of the Asian ancestry and 1.9% of the European ancestry and remained undetected in the African ancestry. Therefore, the rates of somatic mutations in key pathogenic genes involved in NSCLC may have an ancestry-related effect on the mutational spectrum. Because of the historical admixture of the Brazilian population and the patient cohort comprising individuals from different geographic regions, we are aware that the diversity or contribution of the genetic background can only be assessed or even categorized to a limited extent, considering the reduced power of n=10 early-stage patients with co-occurrence of *TP53/EGFR* mutations. As we pointed out, the *TP53* mutation rate in the Brazilian cohort is half of that found in other studies and population cohorts. This query was addressed explanatorily rather than experimentally, as it was not intended to redirect the survey. We did not use molecular tests for ancestry; the patient's ethnicity was based on information from the medical record. However, regarding the *TP53* gene, it is essential to relate it to the miscegenation of the Brazilian population. It is known that Li-Fraumeni syndrome (LFS), caused by the p.R337H variant in the *TP53* gene, is rare in the world population but highly prevalent in the Brazilian population (23). Somatic variant databases rarely describe this variant. However, the identification of this variant in the genomic profile of tumors should be a predictive finding for LFS in the Brazilian population. In fact, one patient in our series was positive for the LFS variant, but patient was not *EGFR*-mutated NSCLC.

In patients with *EGFR*-mutated NSCLC, the coexistence of a *TP53* mutation influenced OS when controlling for age, pathologic stage, relapse, brain metastases, and chemotherapy. This finding suggested that co-mutation is a dependent prognostic marker. Our data contrasted with the report from Labbé et al. (6) who found that concomitant *TP53* mutation status was dissociated from OS in patients with *EGFR*-mutant NSCLC at an early stage who underwent primary surgical resection and received adjuvant chemotherapy. These data indicate that co-mutations were not a strong prognostic marker in early-stage patients. The same study also found that objective response rate is not significantly different between *TP53*-mutant and wild type, and there is a non-significant trend towards shorter OS on *EGFR* with *TP53* mutation in advanced NSCLC patients who received target therapy (6). Therefore, further studies on the utility of *EGFR/TP53* co-mutation as a prognostic and predictive biomarker for early EGFR-mutated NSCLC patients are needed.

Investigations have been conducted to verify whether the type of gene mutation influences the prognostic and predictive effect of *TP53* mutations. Anchored to mutations subtypes, *TP53* mutations showed a remarkable preference for missense mutations over nonsense and frameshift mutations, which are commonly dominant in other tumor suppressor genes such as *RB1* and *PTEN* (24). The study from Labbé et al. (6) showed that NSCLC patients with *TP53* missense mutations have significantly shorter PFS when treated with target therapy. In another published study, *TP53* non-missense mutations reduced responsiveness to target therapy and worsened the prognosis of *EGFR*-mutant advanced NSCLC (25).

Although we demonstrated a predictive and prognostic value of *EGFR/TP53* co-mutations in a small cohort of NSCLC, future validation using a similar cohort with a large set of patients is needed to corroborate the observed correlations. The present study is mainly descriptive and exploratory, and extension of our findings is essential.

Overall, this study presented a significant model for high risk of death and poor OS for patients with stage III NSCLC with relapse and distant metastases, non-responsive to platinum-based chemotherapy and EGFR TKIs, and harboring *EGFR* uncommon mutations, *TP53* mutations, and *EGFR/TP53* co-mutations. Although not currently a therapeutic target, routine inclusion of *TP53* mutation testing by NSG may more accurately determine the effects of this tumor suppressor gene both alone and in combination with other driver mutations in lung cancer and whether there is an interaction with treatment.

### Conclusion

Our study suggested that *TP53* mutation tended to confer poor survival and potential negative predictive effect associated with a non-response to platinum-based chemotherapy and erlotinib in early-stage *EGFR*-mutated resected NSCLC. However, our observation remains to be validated.

## Supplementary Material

Click here to view [pdf].
